# Comparison of diagnostic value of 68 Ga-DOTATOC PET/MRI and standalone MRI for the detection of intracranial meningiomas

**DOI:** 10.1038/s41598-021-87866-9

**Published:** 2021-04-27

**Authors:** Heike C. Einhellig, Eberhard Siebert, Hans-C. Bauknecht, Anna Tietze, Josefine Graef, Christian Furth, Daniel Schulze, Milena Miszczuk, Georg Bohner, Imke Schatka, Marcus R. Makowski

**Affiliations:** 1 Department of Neuroradiology, Charité, Augustenburger Platz 1, 13353 Berlin, Germany; 2 Department of Nuclear Medicine, Charité, Augustenburger Platz 1, 13353 Berlin, Germany; 3 Department of Psychology, Humboldt University, Unter den Linden 6, 10099 Berlin, Germany; 4 Department of Radiology, Charité, Charitéplatz 1, 10117 Berlin, Germany; 5 Department of Radiology, Klinikum Rechts Der Isar, Technical University of Munich, Ismaninger Str. 22, 81675 Munich, Germany

**Keywords:** Neuroscience, Anatomy, Health care, Molecular medicine, Neurology

## Abstract

To evaluate the diagnostic performance of magnetic resonance imaging (MRI) alone in comparison to positron emission tomography/ magnetic resonance imaging (PET/MRI) in patients with meningiomas. 57 patients with a total of 112 meningiomas of the brain were included. PET/MRI, including a fully diagnostic contrast enhanced MRI and PET, were acquired. PET/MRI was used as reference standard. The size and location of meningiomas was recorded. Likelihood-ratio chi-square tests were used to calculate p-values within logistic regression in order to compare different models. A multi-level logistic regression was applied to comply the hierarchical data structure. Multi-level regression adjusts for clustering in data was performed. The majority (n = 103) of meningiomas could be identified based on standard MRI sequences compared to PET/MRI. MRI alone achieved a sensitivity of 95% (95% CI 0.78, 0.99) and specificity of 88% (95% CI 0.58, 0.98). Based on intensity of contrast medium uptake, 97 meningiomas could be diagnosed with intense uptake (93.75%). Sensitivity was lowest with 74% for meningiomas < 0.5 cm^3^, high with 95% for meningiomas > 2cm^3^ and highest with 100% for meningiomas 0.5–1.0 cm^3^. Petroclival meningiomas showed lowest sensitivity with 88% compared to sphenoidal meningiomas with 94% and orbital meningiomas with 100%. Specificity of meningioma diagnostic with MRI was high with 100% for sphenoidal and hemispherical-dural meningiomas and meningiomas with 0.5–1.0 and 1.0–2.0 cm^3^. Overall MRI enables reliable detection of meningiomas compared to PET/MRI. PET/MRI imaging offers highest sensitivity and specificity for small or difficult located meningiomas.

## Introduction

Meningiomas are the most common primary intracranial tumors and represent 36.8% of all primary brain tumors^1^. Indications for meningioma imaging are either periodical observation for asymptomatic meningiomas or treatment stratification (treatment planning or follow up) for symptomatic meningiomas. Close observation is especially recommended for asymptomatic meningiomas with an initial diameter < 2 cm to avoid overtreatment^2^. If treatment is necessary due to symptoms (e.g. seizures), size, growth, edema, location, and/or anatomical relationship of the tumor with the surrounding structures with special focus on health status and age-neurosurgical resection will be method of choice and aims at maximal tumor removal^3,4^. But achieving a complete resection can be challenging.

Besides and additional to neurosurgical resection, other therapy strategies such as radiotherapy (radiosurgery and external fractionated radiotherapy)^5^ or peptide receptor radionuclide therapy (PRRT) exist. Radiotherapy (e.g. radiosurgery, external fractionated radiotherapy) is standard for higher graded meningiomas in post-surgical state (recurrence or residual)^6^ or unresectable meningiomas^7^. PRRT can be performed in recurrent meningiomas with high somatostatin receptor uptake after standard treatments^8^, but further investigation is necessary^9^.

MRI has developed into the imaging technique of choice for assessment and therapy planning of brain meningiomas since it offers an excellent soft tissue contrast and allows for multiplanar imaging with high spatial resolution. Meningiomas can be identified on standard brain MRI, as they appear with isointensity to slight hypointensity on T1 and isointensity to slight hyperintensity on T2 images relative to grey matter^10^. Additional features such as homogenous contrast medium uptake or typical MR-signs (dural tail sign, broad-based dural attachment, displacement of grey matter, displaced subarachnoidal space, calcifications or CSF leakage) are helpful for a reliable diagnosis^11^. But especially for diagnosis of tumor recurrence structural imaging techniques have been limited in delineating meningiomas-dependent on location (e.g. skull base), surrounding involvement (e.g. bone) or tumors with special geometry^12^. In addition, differentiation between viable tumor from scar tissue or post-therapeutic changes by MRI alone-particularly after radiotherapy can be challenging.

Therefore, molecular imaging modality using PET imaging with somatostatin receptor (SSTR) ligands such as 68 Ga—DOTATOC or—DOTATATE were recommended to guide clinicians from all disciplines involved in the management of patients with meningiomas in the diagnosis and follow-up^13^. PET/MRI with 68 Ga-DOTATOC as somatostatin receptor radiopharmaceutical has been shown to provide an ideal combination of high sensitivity/specificity (PET) and the best possible morphological visualization of meningiomas (MRI)^1^, all of which are of potential clinical benefit to patients. But as a result of high costs and low availability this technique is not available to all patients.

The aim of our study was to evaluate the diagnostic performance of standard MRI imaging in comparison to hybrid imaging with positron emission tomography/ magnetic resonance imaging (PET/MRI) in patients with meningiomas.

## Material and methods

### Study sample

We confirm that all methods were carried out in accordance with relevant guidelines and regulations. Additionally we confirm that all experimental protocols were approved by the local ethics committee: institutional review board approval from Ethics Committee of Hospital affiliated to Charité University Medicine (Berlin, Germany) was obtained. Written informed consent was obtained from all subjects. From March 2017 to December 2019, 68 patients with image-based diagnosed meningioma suspicious lesions were referred to our department for PET/MRI imaging and were consecutively included in our study. Following cases were excluded due to clinical exclusion diagnostics (prior MRI untypical for meningioma): non-classifiable tumor (n = 3), pituitary adenoma (n = 3), prolactinoma (n = 1), optic neuritis (n = 1), IgG-syndrome (n = 1), tolosa hunt syndrome (n = 1), inflammation (n = 1) and glomus tumor (n = 1). In total 112 meningiomas in 57 patients were diagnosed based on PET/MRI imaging (mean age 58.2 ± 15.2 years, age range 37–84 years; 18 males: mean age 54.9 ± 14.4 years, age range 27 to 77 years and 39 females: mean age 59.5 ± 15.4 years, age range 34 to 84 years; difference in mean age values between males and females: p = 0.356). 39 patients underwent surgery with known positive histological report in 32 cases (WHO I: 17x; WHO II: 13x; WHO III: 2x). PET/MRI and conventional brain MRI were performed in all patients.

### Imaging protocol

PET brain scans and MRI brain scans were performed simultaneously using a standardized imaging protocol on a 3-T PET/MRI scanner (Biograph mMR, Siemens Healthcare GmbH, Wittelsbacherpl. 1, 80333 München); equipped with a commercially available brain coil. 68 Ga-DOTATOC (gallium-68–labeled [DOTA0-Phe1-Tyr3] octreotide) as radiopharmaceutical-used for the PET brain scan-was injected intravenously (mean 163.2 MBq; interquartile range [IQR] 154.3–168.0 MBq). After intravenous administration of 68 Ga-DOTATOC the PET scan was performed with the patient in supine position (at a median time of 77.9 (IQR 60–80 min) minutes). PET brain scan included the whole skull (single bed position, 20 min’ duration, 3D list mode acquisition). Ordered subset expectation maximization (OSEM) algorithm was used for PET raw data reconstruction (subsets 3, iterations 3, voxel size 1.04 × 1.04 × 2.03 mm^3^, image matrix 344 × 344 × 127). Images were filtered using a 3D Gaussian filter (3-mm). Attenuation and scatter correction were performed on ultra-short echo time sequence (UTE).

For MRI brain scans standard brain MR-imaging-sequences were obtained: axial T2 turbo spin echo-weighted with fat saturation sequences (T2 TSE fs), axial T1-weighted FL2D sequences (T1 FL2D), axial susceptibility-weighted sequences (SWI), axial diffusion-weighted with apparent diffusion coefficient maps (DWI/ADC; EP2D), coronal turbo inversion recovery magnitude (TIRM) sequences without contrast medium. After contrast injection contrast-enhanced isotropic 3D high-resolution T1-weighted sequences magnetization-prepared rapid acquisition with gradient echo (MP-RAGE) sequence with coronal and sagittal reconstruction and axial T1-weighted FL2D were acquired in all patients. For contrast enhanced T1-weighted imaging a body-weight–adjusted dose of a gadolinium-based contrast agent (Gadovist, Bayer Healthcare, Kaiser-Wilhelm-Allee 1, 51373 Leverkusen) was injected intravenously, followed by a saline flush. In addition, T1- and T2-weighted sequences of the optic tract with post contrast axial T1 StarVIBE were acquired in selected patients. Table 1 shows the imaging protocol of the MRI.

**Table 1:** Parameters of the imaging protocol of the brain and the optical nerves.

Sequence	T2 TSE FS	EP2D DWI	T2 TIRM DARK FLUID	SWI	T1 FL2D	T1 FL2D ( +)	MPR age ( +)	T1 STAR VIBE ( +) orbital	T1 TSE FS ( +) orbital
Scan plane	Axial	Axial	Coronal	Axial	Axial	Axial	Sagittal	Axial	Coronal
Voxel size, mm	0.4 × 0.4 × 3.0	1.2 × 1.2 × 3.0	0.0 × 0.4 × 4.0	0.0 × 0.9 × 1.0	0.7 × 0.7 × 5.0	0.7 × 0.7 × 5.0	1.0 × 1.0 × 1.0	0.8 × 0.8 × 1.0	0.3 × 0.3 × 2.0
No. slices	48	48	38	120	30	30	192	64	24
TR/TE, ms	5320/88	10,000/101	8000/94	27/20	250/2.66	250/2.66	2400/2.66	5.0/2.07	719.0/9.1
Averages	2	4	1	1	2	2	1	2	3
FoV, mm	230	230	230	220	230	230	256	150	150
Flip angle, degrees	150		150	15	70	70	8	9.0	120
Matrix	Auto (triple)	Auto (triple)	Auto (triple)	Auto (triple)	Auto (triple)	Auto (triple)	Auto (triple)	Auto (triple)	Auto (triple)

All sequences without contrast medium except marked with (+).

### Imaging analysis

One radiologist with additional nuclear medicine specialty (more than 5 year experience with radiology, 4 year experience with nuclear medicine (including PET/MRI brain) and 1 year experience with neuroradiology) reviewed all MR and all PET/MRI images independently in a randomized fashion. The observer analyzed the PET/MRI and MRI scans using Visage 7.1 (Visage Imaging GmbH, Lepsiusstraße 70, Berlin, Germany) for quantitative and visual analysis due to daily experience. The reader was blinded to the results of other diagnostic procedures, patient identity and the clinical history of the patients. For the evaluation of PET/MRI, the observer used all anonymized information given by fusioned PET/MRI images, MRI sequences and PET data. For MRI only evaluation, the observer used information given by all examined MRI sequences 5 months later. The time gap of 5 months was chosen to eliminate risk of bias due to recognition of individual patients and their meningiomas. The observer did not experience any memorability.

For the diagnosis of meningiomas, PET/MRI studies including the imaging modalities (MRI, 68 Ga-PET) of the patients were taken as gold standard. For image-based diagnoses of meningioma, MRI- and PET criteria were taken into consideration. The MRI part was based on the following properties: iso- to hyperintense in T1 weighted and iso- to hypointense in T2 weighted images, intensity and homogeneity of contrast uptake such as MR signs. Typical MR signs were considered dural tail sign, broad-based dural attachment, displacement of grey matter, displaced subarachnoidal space, calcifications and/or CSF leakage.

For PET images standardized uptake values (SUV) for body weight were measured and visually analyzed dependent on intensity of uptake compared to background. For PET data quantification for pathological 68 Ga-DOTATOC uptake, a three-dimensional region of interest (3D ROI) was defined using maximum, mean and minimum standardized uptake values (SUV_max_, SUV_mean_ and SUV_min_). The greatest extent and the SUV values of the respective lesion were recorded in the transaxial, attenuation-corrected PET-slice. Regions of interest and volume were manually defined in 3D mode using automatic standard software tool avoiding the periphery of lesions to minimize partial volume effects. SUV values of the healthy brain tissue contralateral to the lesion was measured with a 2D ROI (1 cm diameter).

For evaluation of PET/MRI, the PET Data were first registered to the post contrast T1-weighted MR images using the rigid registration algorithm residing on Osirix. The resulting transformation matrix was then applied to the PET image set to register it to the MRI images.

**Figure 1:**
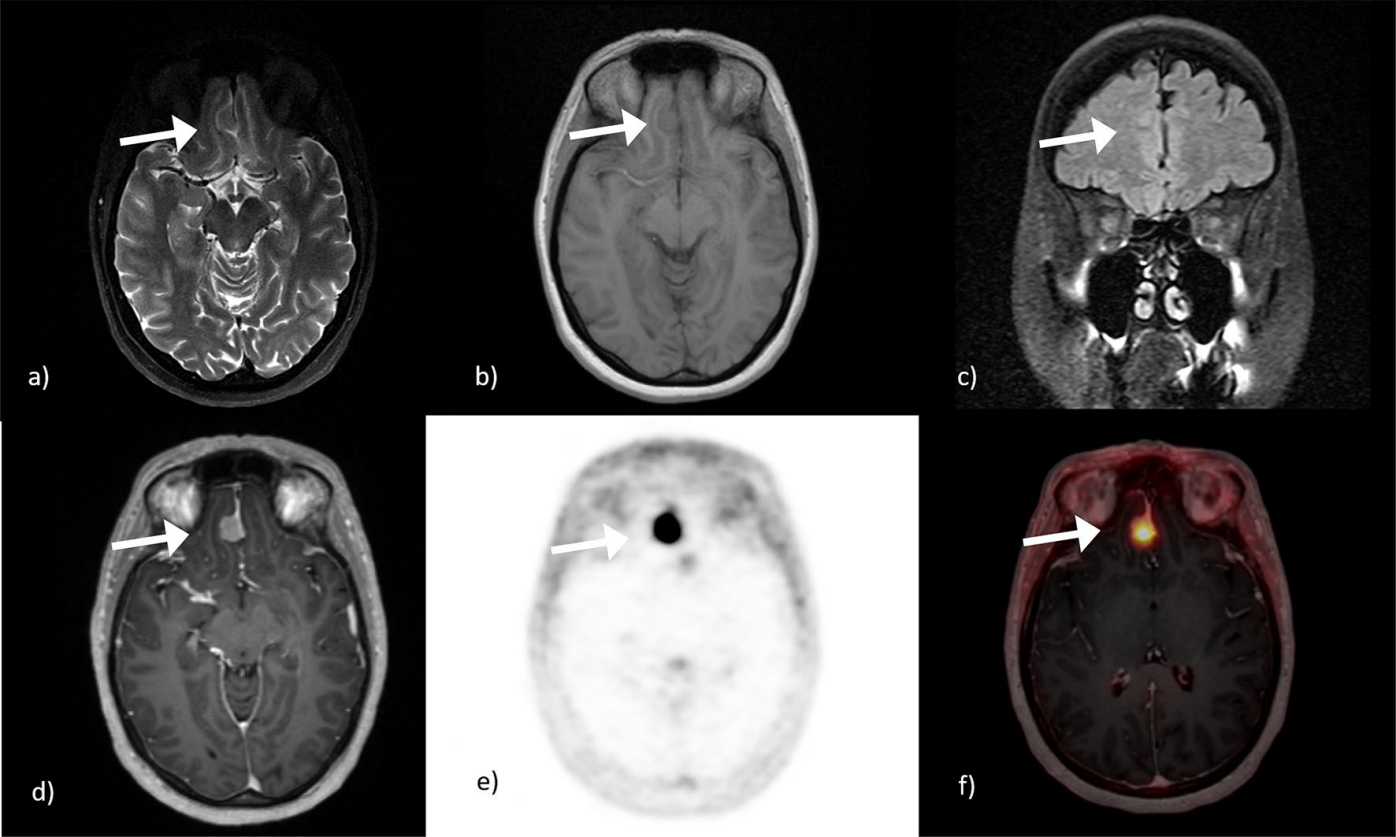
61 years old female patient with a typical falcine meningioma in the front. The first row presents the difficulty in differentiation the meningioma from the surrounding parenchyma due to isointensity. Here presented for (**a**) T2 TSE with CSF cleft sign, (**b**) T1 FL2D and (**c**) TIRM without edema. However the falcine meningioma can be easily detected after contrast medium injection in MPRage (**d**) after 68 Ga-DOTATOC injection for PET (**e**) and in fusioned imaging PET/MRI (**f**).

**Figure 2:**
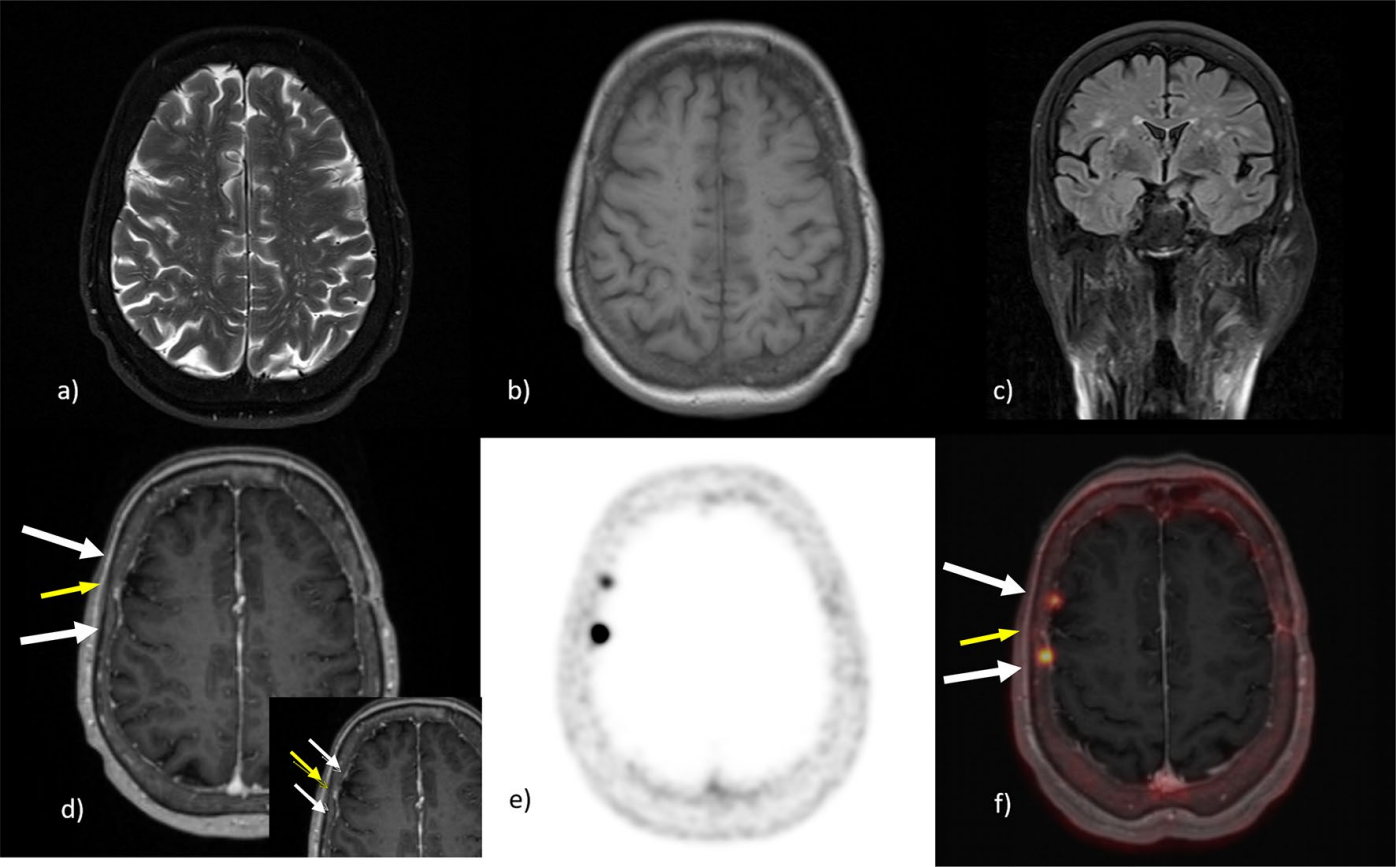
76 years old female patient with two small frontal ‘en plaque’ meningiomas (white arrows). Very close focus is necessary to distinguish the vessel in the center (yellow arrow) from the two surrounding meningiomas. The meningiomas were non-detected due to their small sizes (**a**) T2 TSE, (**b**) T1 FL2D and (**c**) TIRM. But they could have been detected in MPRage (**d**) the ‘en plaque’ meningiomas are clearly detectable in (**e**) PET und (**f**) PET/MRI fusion.

See Figs. 1 and 2 for imaging examples.

### Statistical analysis

We used logistic regression in order to assess the diagnostic quality of MRI and PET in contrast to our gold standard method (PET-MRI). The fit of a logistic regression model corresponds to the overall predictive accuracy and is as such related to comparable analysis techniques like ROC analysis. We utilized likelihood-ratio chi-square tests to calculate p-values within logistic regression in order to compare different models. Sensitivity and specificity were calculated from logistic regression^14^. More specifically, we applied multi-level logistic regression to comply with the hierarchical data structure (124 meningiomas within 68 patients)^15^. Multi-level regression adjusts for clustering in data, i.e., detecting a meningioma is more likely if the patient has another already detected meningioma. In subgroup analysis, we dropped multi-level analysis due to sparse data.

## Results

### Patient measurements

**Figure 3:**
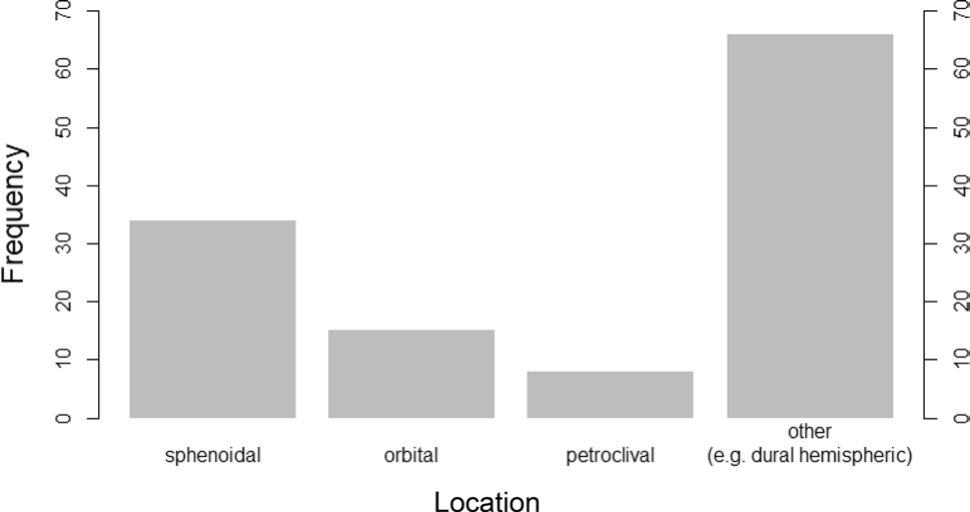
Overview of meningioma location. Frequency of meningiomas in typical locations. The majority of meningiomas were found hemispherical dural. The second most location was sphenoidal. The third most prevalent location was along the N. opticus in the orbit. Petroclival meningiomas were rare.

A total of 68 patients with 124 lesions underwent a PET/MRI examination due to suspicion/exclusion for meningioma and were therefore included in our study. 57 patients with 112 PET/MRI-based diagnosed meningioma were included in our study. The mean age of all patients was 57.1 years. 31.6% were male and 68.4% were female patients. Meningiomas were located in different but typical locations (Fig. 3).

**Figure 4:**
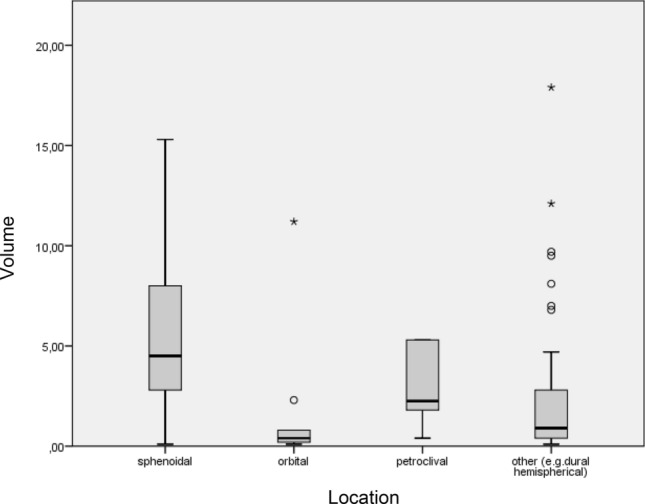
Volume measurements dependent on location. Size measurements in MRI were shown highest values for sphenoidal meningiomas. The highest measurement is 48.4 cm^3^ was sphenoidal (not included in the diagram due to scale reasons). The smallest sizes were measured for optical sheat meningiomas.

In total meningiomas had an average size of 5.2 cm^3^. The biggest meningiomas in average were measured for the sphenoid region (mean 10.8 cm^3)^. The smallest meningiomas were measured for the orbit location (mean 2.2 cm^3^) (see Fig. 4).

### Meningioma results in MRI

103 out of 112 meningiomas, ranging from 0.1 to 48.4 cm^3^, could reliably be detected by MRI.

#### Visual imaging characteristics in MRI

**Figure 5:**
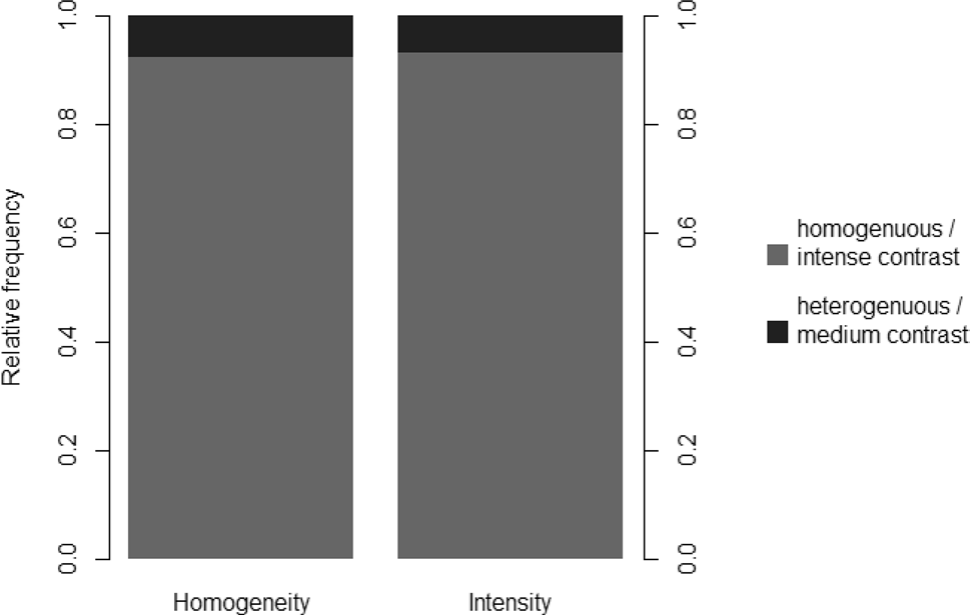
Intensity and homogeneity of contrast medium uptake. All meningiomas showed typical contrast enhancement features with at least medium but way dominant intensive contrast enhancement. Moreover the contrast enhancement was almost always homogeneous as expected. The intensity and homogeneity were the most helpful image feature for the readers.

All MRI-detected meningiomas (n = 103, 100%) were mainly diagnosed due to contrast enhancement performance (Fig. 3): especially high (93.6%) and medium (6.4%) intensity and homogeneity (94.4%) have proven to be a helpful marker (Fig. 5).

**Figure 6:**
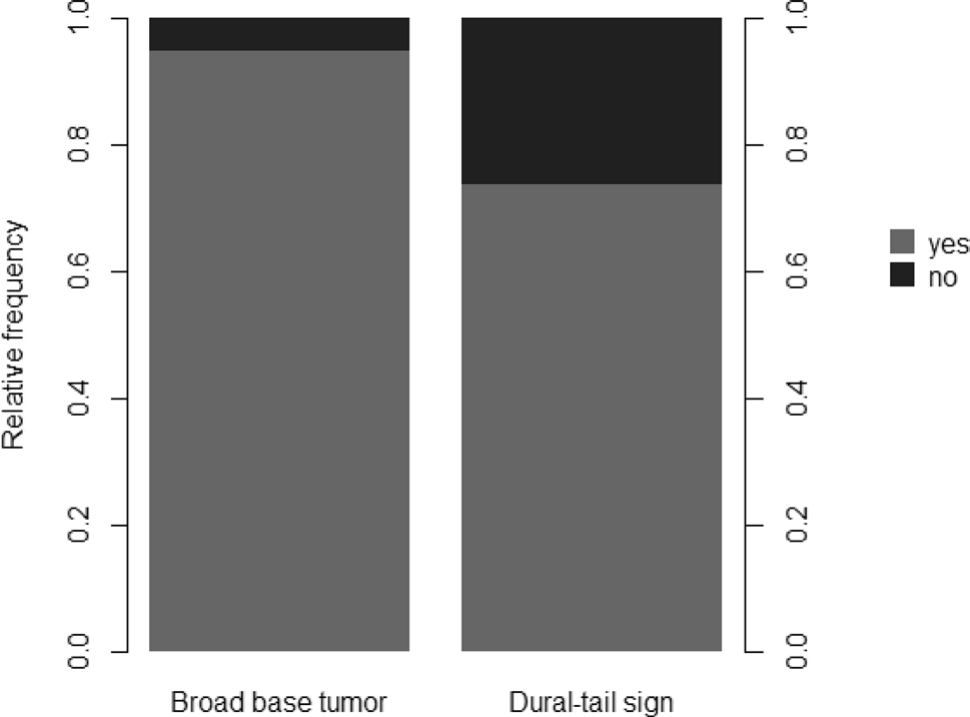
Broad-based tumor and dural tail sign as meningioma MRI-characteristics. Typical structural aspects for meningioma were mainly and easily identified based dural tail sign and its broad base. Other features such as hypointensity in T1 or hyperintensity in T2 to grey matter or calcifications were less often.

In addition, MRI signs such as dural tail sign (78.8%) and broad base (97.1%) of meningiomas have been the most recurring markers in our evaluation of meningioma criteria. Whereas signs like the CSF cleft sign have not been helpful (39.4%) (Fig. 6).

#### Sensitivity and specificity of MRI

Overall the sensitivity of MRI for the detection of meningiomas was high (0.95; 95% CI 0.78, 0.99). The same accounted for specificity (0.88; 95% CI 0.58, 0.98). Both were statistically significant as zero was not part of the intervals (Table 2).

Location: Regarding the location of meningiomas, MRI only showed highest sensitivity of 1.0 for the orbital region (95% CI 0.19–0.99). The sphenoidal region showed sensitivity of 0.94 (95% CI 0.85–1.00). No false positive cases occurred, thus specificity was 1.0 (CI not defined). Regarding the orbital location, the sensitivity was measured 1.00, as we did not obtain any false negatives. Sensitivity of 0.88 was measured for other (e.g. dural; 95% CI 0.79–0.96) and petroclival (95% CI 0.63–1.00) region. Table 2 represents sensitivity and specificity of MRI only corresponding to location.

Size: A small number (n = 9; 8%) of meningiomas were not detected in MRI (mean volume 0.51 ± 0.5 cm^3^). MRI-based sensitivity for meningiomas < 0.5 cm^3^ was 0.74 (95% CI 0.55–0.93). Meningiomas with a volume 0.5–1.0 cm^3^ were all diagnosed via MRI only (sensitivity of 1.00). Sensitivity of meningiomas with volume 1.0–2.0 cm^3^ was measured 0.93 (95% CI 0.79–1.00) and 0.95 (95% CI 0.90–1.00) for volumes > 2cm^3^. Further details (see Table 2).

MRI alone was significantly able to predict the results from PET-MRI (LRT: χ^2^(1) = 26.90, p < 0.001). The resulting sensitivity and specificity were high accordingly. When looking into the dependence of sensitivities on the volume of the lesion (Mdn = 2.5cm^3^, Min = 0.10cm^3^, Max = 75.8cm^3^), we found no statistically significant effect (interaction of predictive accuracy and volume in cm^3^: p = 0.55).

**Table 2:** The table demonstrates the overview of specificity and sensitivity dependent on location and size for MRI.

	Sensitivity	95% CI	Specificity	95% CI
Overall	0.94	0.74–0.99	0.93	0.67–0.99
Location: sphenoidal	0.94	0.85–1.00	1.00	–^a^
Location: orbital	1.00	–^a^	0.75	0.26–1.00
Location: petroclival	0.88	0.63–1.00	–^b^	–^b^
Location: other	0.88	0.79–0.96	1.00	–^a^
Volume < 0.5 cm^3^	0.74	0.55–0.93	–^b^	–^b^
Volume 0.5–1.0 cm^3^	1.00	–^a^	1.00	–^a^
Volume 1.01–2.0 cm^3^	0.93	0.79–1.00	1.00	–^a^
Volume > 2 cm^3^	0.95	0.90–1.00	0.75	0.26–1.00

^a^Lack of information due to perfect fit.

^b^Lack of information due to sparse data.

### Meningioma results in PET

#### Visual imaging characteristics in PET

Meningiomas have shown a visual and measurable increased tracer uptake (mean SUV_max_ 10.8 ± 10.3, mean SUV_mean_ 4.1 ± 3.1). For MRI non-detected meningiomas the uptake was lower (mean SUV_max_ 7.4 ± 7.1, mean SUV_mean_ 2.4 ± 1.4). The differences for SUV-values were not significant (p_SUVmax_ = 0.459, p_SUVmean_ = 0.132).

#### Sensitivity and specificity of PET

Overall the sensitivity was very high (0.99; 95% CI 0.94, 1.00), as well as specificity (0.83; 95% CI 0.41, 0.97), and significantly different from zero.

Location & size in PET: Regarding the sphenoidal location, the sensitivity was 1.00, as we did not obtain any false negatives (CI not defined) with a specificity of 0.75.

Regarding the orbital location, the sensitivity and specificity both were 1.00, as PET-MRI and PET agreed perfectly with orbital meningioma.

PET however slightly overestimated the size of meningiomas compared to MRI (PET 5.8 ± 10.1 vs 4.6 ± 9.3 cm^3^; p < 0.05).

## Discussion

MRI demonstrates a comparable sensitivity and specificity to PET/MRI for the detection of meningiomas. MRI can reliably be used for meningioma treatment planning and follow up as treatment procedures for clinicians, if no PET/MRI is available. MRI is—considering the overall patient situation—a supportive tool for meningioma treatment primarily starting at sizes of more than 2 cm. Consequently they can reliable use pure MRI as planning und follow up tool. Moreover PET/MRI is especially helpful for the detection of small meningiomas at challenging locations compared to MRI alone.

### Current clinical assessment of PET/MRI and MRI

68-Ga-DOTA PET/MRI as hybrid imaging is described to have the combination of high sensitivity and high specificity and the best possible morphological visualization of meningiomas^1,5^. Dependent on treatment strategy (radiotherapy vs. neurosurgical resection) different diagnostic information is required.

MRI is imaging method of choice related to its excellent soft-tissue contrast and high spatial resolution^3^. It is best in evaluation of exact extent, compression of structures (e.g. vessels or nerves) and is nowadays relatively easily available with a medium cost effort. However, MRI’s specificity for tumor tissue is lower, resulting in challenges for clinicians. Therefore it can be challenging to determine meningiomas especially for smaller sizes due to the limitations of structural imaging or for the distinction between scar vs recurrence^16–20^.

PET/MR imaging is the least available but most cost intense imaging modality for meningiomas due to 68 Ga-SSTR (e.g. DOTATOC or DOTATATE) production compared to contrast enhanced MRI imaging. Although automated PET radiopharmaceutical synthesis systems with increased reliability, reproducibility and safety are available due to in-house production with a 68Ge/68 Ga generator system without need of an on-site cyclotron, still technical and radiopharmaceutical procedures can be error-prone resulting in short-term appointment cancelling. Most notably with focus on the physical half-life time of 68-Gallium (68 min). One strength of PET imaging is its high sensitivity, what allows the detection of even picomolecular amounts of radiotracer. But spatial resolution is the weakest point of PET imaging-despite the modern technological evolution of PET imaging.

However-regarding diagnostic accuracy-PET imaging developed to be the method of choice especially for radiotherapists: in delineating meningioma tissue even in post-therapeutic state in terms, in definition of gross target volume (GTV) and clinical target volume (CTV)^21,22^ and benefits non-experienced radiotherapists^23^. But also for neurosurgeons, the high tumor-to-background ratio, along with the advent of hybrid PET and MRI system, with its highly sensitive and specific diagnosis of intracranial meningiomas is very helpful^24^. Due to the expression of somatostatin receptor 2 meningiomas meningioma tissue can be easily delineated from healthy or scar tissue by 68 Ga-DOTATATE as PET tracer^25^. Moreover it is useful in cases of unclear differential diagnosis between tumor progression and post-therapeutic reactive changes or in cases of detecting meningiomas not (yet) seen in MRI.

For skull base or cavernous sinus located meningiomas or meningiomas with transosseous extension, PET imaging with e.g. DOTATATE and DOTATOC PET was superior to MRI regarding tumor delineation^26–28^. In addition DOTATATE PET can be helpful to differentiate optic nerve sheath meningiomas from other non-tumoral optic nerve affecting lesions^29^.

These are the reasons why the RANO/PET group provided recommendations for the use of PET imaging in the clinical management of meningiomas^30^.

### MRI for the assessments of meningiomas

The results of our study confirmed that MRI allows a reliable identification of meningiomas with focus on contrast enhanced images together with typical MRI signs. A former study-comparing contrast-enhanced MRI and 68 Ga-DOTATOC PET/CT prior to radiotherapy treatment-showed, that only 90% of meningiomas were detected by contrast-enhanced MRI consecutively indicating a better sensitivity for DOTATOC PET/CT in detection of meningiomas^31^.

Additionally, our study suggest, that according to sensitivity of 74%-MRI enables reliable detection of small focal meningiomas with special focus of an experienced reader. However enfeebled by reliable visualization in standard MRI brain sequences due to partial volume effect. Even for difficult locations (e.g. skull base and orbit), meningiomas can be seen and correctly interpreted dependent on diagnostic reader experience.

### PET for the assessment of meningiomas

In our study, all meningiomas detected on PET could be identified as meningiomas using a combination of PET and MRI. PET allowed the identification of meningiomas with a high intraobserver agreement—but only little higher sensitivity-compared to standard MR brain sequences. Additionally, our study suggests that PET enables the detection of very small focal meningiomas due to its high meningioma/background ratio^32^, which cannot reliably be detected using standard MRI brain sequences.

Analysing the combined information from PET/MRI, meningiomas could be reliably detected in standard brain MRI images with contrast enhanced MPRage sequences and their typical MRI signs such as dural tail sign and broad based tumors. It can be challenging to keep focus on very small but detectable lesions. However, very small meningiomas (e.g. en plaque meningiomas) are visible and are mainly overlooked according to reader’s experience. Taking advantage of these properties, MRI is a powerful image tool for the detection, follow up or treatment planning of meningiomas due to almost equal sensitivity compared to PET/MRI. While MRI size-measurements are quite reliable, PET slightly overestimated the size of meningiomas. This can be explained by the lower resolution of PET compared to MRI. Especially in menigiomas the attenuation correction has an impact and may lead to overestimation of size. Moreover disadvantage of gallium-68 may be compromised spatial resolution due to a relatively high positron energy and thus relatively long positron range^33^.

### Limitations

Our study has a number of potential limitations. Potential risk of bias due to one experienced reader. This study does not evaluate the experience of an untrained reader. An incomplete histological analysis of meningiomas was performed. PET/MRI was used as the gold standard with image-based diagnoses without complete histological confirmation. Our study does not include an analysis how findings by MRI influence clinical management or clinical outcome of patients. Findings in MRI were not correlated with the symptoms of patients. Further studies are needed to evaluate the potential of MRI in a larger patient collective.

### Future studies

First-line treatment of symptomatic patients is usually based on surgery. It is generally agreed that a considerable proportion of cases in patients with treatment compared to non-treated patients may influence the results. In these cases it is of clinical importance to know the exact contrast enhancement due to postsurgical state. In addition, different histological subtypes may have different contrast enhancement or tracer uptake effects. Moreover, other sequences such as SWI can be interesting due to the fact that meningiomas are mainly diagnosed in CCTs due to their calcifications. In future studies it could be assessed, whether a taller number of meningiomas and patients could help to underline our results.

## Conclusion

PET/MRI is especially helpful for the detection of small meningiomas or at challenging locations compared to MRI alone. MRI alone demonstrates a comparable sensitivity and specificity to PET/MRI for the detection of clinical-relevant meningiomas larger than 2 cm.

## Abbreviations

PET: Positron emission tomography
MRI: Magnetic resonance imaging
DOTATOC: DOTA-D Phe1-Tyr3-octreotide
Ga/Ge: Gallium/germanium
PRRT: Peptide receptor radionuclide therapy
UTE: Ultra-short echo time
TSE fs: Turbo spin echo-weighted with fat saturation
T1 FL2D: T1-weighted Flash 2-dimensional
SWI: Susceptibility-weighted imaging
DWI: Diffusion-weighted imaging
EP2D: Echo-planar imaging 2-dimensional
ADC: Apparent diffusion coefficient
TIRM: Turbo inversion recovery magnitude
MP-RAGE: Magnetization-prepared rapid acquisition with gradient echo
VIBE: Volumetric interpolated breath-hold examination
SUV: Standardized uptake values
Max/Min: Maximal/minimal
ROC: Receiver operating characteristic
CI: Confidence interval
GTV: Gross target volume
CTV: Clinical target volume
DOTATATE: DOTA-0-Tyr3-octreotate
ROI: Region of interest
